# Nanoparticle-assisted strategies in mass spectrometry-based metabolite analysis: recent advances across analytical workflow

**DOI:** 10.1007/s00604-025-07473-7

**Published:** 2025-08-23

**Authors:** Roberto Gamboa-Becerra, Ernesto Beltrán-Partida, Benjamín Valdez-Salas, Jhonathan Castillo-Saenz, Jorge Salvador-Carlos, Mario Curiel-Álvarez

**Affiliations:** Core Facilities of Chemistry and Advanced Materials, Instituto de Ingeniería, Universidad Autónoma de Baja California, Calle de La Normal S/N and Boulevard Benito Juárez, Mexicali, 21100 Baja California Mexico

**Keywords:** Sample pretreatment, Selective metabolite enrichment, Targeted metabolite derivatization, Detection sensitivity enhancement, Analytical techniques, Nanoparticle-assisted metabolomics, Analytical workflow optimization

## Abstract

Metabolite analysis plays a critical role in understanding phenotypic variations, biochemical processes, and physiological responses in biological systems. Whether through untargeted metabolomic profiling or targeted approaches aimed at quantifying specific or even individual metabolites, accurate detection presents significant analytical challenges due to their vast chemical diversity, low abundance, and complexity of biological matrices. This chemical analytical process encompasses a dynamic workflow that includes sample collection, extraction, enrichment, separation, and detection. Recent advances in nanotechnology offer promising alternatives to support and enhance each stage of this workflow, particularly within mass spectrometry (MS)-based applications. Nanoparticles, due to their high surface area, tunable surface chemistry, and ability to improve sensitivity, have been widely applied to improve sample pretreatment, selective enrichment, separation efficiency, and ionization, ultimately enhancing MS-based metabolites detection. This review provides an updated overview of nanoparticle-assisted strategies throughout the MS-based metabolite analysis workflow. It discusses the different classes of those nanomaterials and their applications across various phases stages, from sample preparation to ionization and detection, supporting analyses that range from untargeted and targeted metabolomics to the detection of individual metabolites. Although the primary focus is on MS-based workflows, we also reviewed nanoparticle-assisted separation strategies coupled with alternative detection platforms, such as optical or electrochemical methods, when these approaches show potential for integration with MS workflows. This inclusion reflects the current gap in literature addressing nanoparticle-assisted separation directly coupled with MS detection systems. These cases highlight underexplored opportunities where nanomaterials could enhance separation prior to MS detection, although further work is needed to ensure compatibility with MS platforms for suitable metabolite analysis. Furthermore, we highlight emerging trends and future perspectives in this evolving field, emphasizing the potential of nanotechnology to overcome current analytical limitations and expand the scope of both metabolomic profiling and focused metabolite analysis.

## Introduction

Metabolite analysis plays a vital role in characterizing small molecules in biological systems, supporting diverse areas of metabolism research. This field has advanced through high-throughput, sensitive analytical techniques, such as mass spectrometry (MS), which enable metabolite detection at the systems level [1]. Metabolomics, a key subfield, aims to characterize metabolites with molecular weights under 1500 Da in biological systems, such as cell lysates, biofluids, tissues, or entire organisms, and which presents significant analytical challenges [2, 3]. Trough MS and high-throughput analytical techniques, metabolomics provides powerful insights into phenotypes across diverse disciplines, such as agriculture, clinical research, drug development, disease diagnosis and treatment, nutrition, biomarker discovery, and environmental science [4, 5].

It offers a systems-wide view of the metabolic complexity, diversity, and interconnectivity of biochemical pathways, facilitating the generation of new hypotheses [6].

Metabolite analysis presents challenges for correct and accurate molecular determination. First, only ~ 7% of an organism’s composition constitutes is estimated to be metabolites [7]. Second, the immense chemical diversity of metabolites whit significant variations in physicochemical properties, such as molecular weight, chemical structure, solubility, hydrophobicity, polarity, and ionization efficiency, must be considered. Third, the wide dynamic range of metabolite concentrations further complicates the analysis [8]. These factors can significantly influence the detection and accurate quantification of metabolites across different analytical techniques. To address these, chromatographic methods such as gas chromatography, liquid chromatography (LC), and capillary electrophoresis, are often combined with MS to minimize peak overlap, ion suppression, and enhance both qualitative and quantitative analysis of metabolites [9].

Metabolomics has shown the potential to measure a vast number of metabolites in a single biological sample with relatively simple sample preparation and minimal physical separation steps. However, challenges persist, such as removing background signals from proteins and biomacromolecules, signal enhancement or suppression, simplifying overloaded spectra, achieving uniform ionization across metabolites, and distinguishing overlapping signals belonging to the same molecule [7]. This underscores the need for comprehensive and adaptable analytical workflows.

MS-based workflows for metabolite analysis encompasses sample collection, extraction, enrichment, separation, and detection. Depending on the aim, these workflows range from broad untargeted metabolomics to targeted analysis, and additionally, highly sensitive quantitative strategies are to precise analysis of a few or even single metabolites. Each approach presents challenges metabolite diversity, low abundance, and matrix complexity [10, 11]. In this context, nanotechnology offers promising tools to improve various stages of the MS-based workflow.

**Fig. 1:**
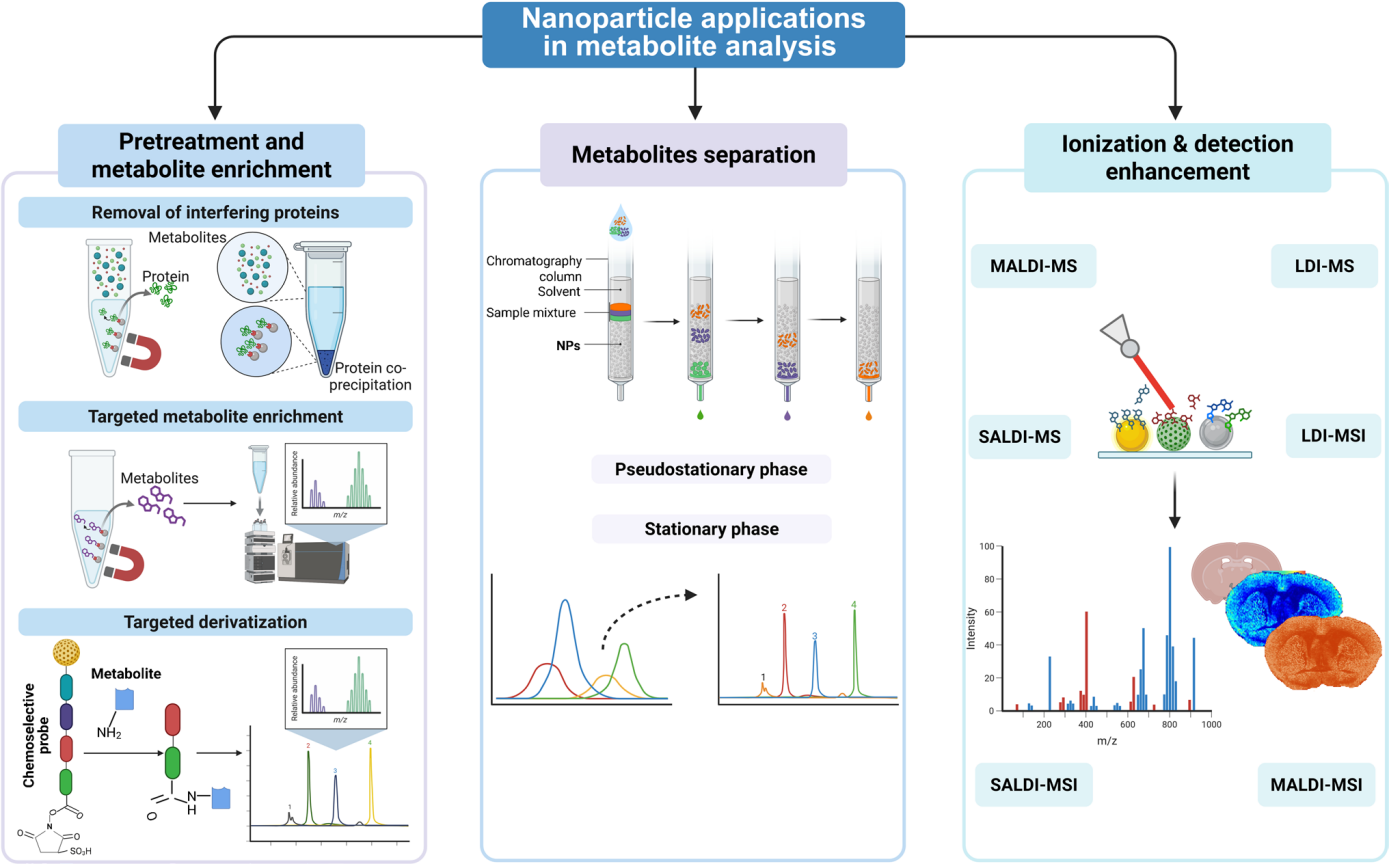
Nanoparticles applications in metabolite analysis workflow

Recent advancements in nanotechnology have improved metabolite analysis, particularly in sample preparation and MS detection (Fig. 1). Nanoparticles (NPs), with three spatial dimensions, typically sized from 1 to 100 nm in at least one dimension, possess high surface area, biocompatibility, and distinctive optical, thermal, electrical, and magnetic properties [12], which make them valuable for applications in drug delivery, catalysis, imaging, and biochemical analysis. In metabolite analysis workflows, including but not limited to metabolomics, NPs can enhance selectivity, sensitivity, and separation efficiency. They serve as enrichment sorbents, or chemo-selective probes in sample pretreatment, as stationary or pseudo-stationary phases in chromatographic separation, and as matrices for MS detection [13, 14].

NP-assisted MS has gained attention for its utility in enhancing metabolite analysis workflow efficiency. Reviews by Zhang et al. [7] and later by Li et al. [13] highlight NP roles in metabolite analysis workflows, in sample preparation, analyte separation, and detection. In this review, we considered NPs not only as individual units but also as components of core–shell structures, hybrid nanomaterials, and composites for their synergistic effects on analytical performance. We focused on NP-enhanced MS workflows in both untargeted and targeted metabolomics, as well as analyses of selected or single metabolites. Additionally, emerging trends and future perspectives in nanoparticle-enable MS workflows for metabolite analysis are discussed.

## Nanoparticles for sample pretreatment and metabolite enrichment

In sample pretreatment, the selective properties of nanoparticles (e.g., high affinity, electrostatic interactions), initially considered for the removal of interfering proteins, have been more effectively exploited for the selective capture and enrichment of low-abundance metabolites, as well as for targeted derivatization to enhance metabolite detectability [15]. Different types of nanoparticles used in these applications include metal oxides, magnetic nanoparticles (MNPs), metal–organic frameworks (MOFs), covalent-organic frameworks (COFs), molecularly imprinted polymers (MIPs), silica (SiO_2_) nanoparticles, and carbon-based nanomaterials (Table 1).

**Table 1:** Applications of nanoparticle-based strategies in biological samples pretreatment for selective metabolite enrichment

Nanoparticles	Class	Metabolites	Size (nm)	LOD	Applied modes	Analytical instrument	Sample matrix	Reference
Fe_3_O_4_@SiO_2_-C18	MNPs	Pyrethroid pesticides	100	0.001–0.008 μg/L	MSPE	GC–MS	Water	[24]
Fe_3_O_4_@SiO_2_@PEI-FPBA	MNPs	Nucleosides	32	0.2–3.0 μg/kg	MSPE	UHPLC-MS/MS	Human urine	[21]
Fe_3_O_4_@PEI-FPBA	MNPs	Catecholamines	35–40	-	MSPE	UHPLC-MS/MS	Human urine	[23]
Fe_3_O_4_@SiO_2_-C18	MNPs	VOCs	10	9.7–57.3 ng/mL	MSPE	GC–MS	Human urine	[25]
Fe_3_O_4_@PEI@TBA	MNPs	Ginsenosides	20–30	-	MSPE	UHPLC-MS	Rat plasma	[22]
Fe_3_O_4_@SiO_2_@C18	MNPs	Pesticides	8.4	1.9–62 ng/L	MSPE	GC–MS	Water	[26]
Au	Metallic NPs	Oxidized phospholipids	28	0.11–0.26 ng/mL	d-SPE	HPLC–MS/MS	Human plasma	[29]
TiO_2_ − ZrO_2_/SiO_2_	Metal oxides NPs	Nucleosides	TiO_2_ − ZrO_2_ NPs: 1–2	1–2	d-SPE	HPLC–MS/MS	Human urine	[20]
MOF-5	Magnetic MOFs	PAHs and GAs	Fe_3_O_4_ NPs: 30–50	PAHs: 0.91–1.96 ng/L GAs: 6–80 ng/L	MSPE	GC–MS	Soil, fish, and plant samples	[32]
Fe3O4@SiO_2_@MOF	Magnetic MOFs	Domoic acid	Fe3O4 NPs: < 100	1.45 pg/mL	MSPE	HPLC–MS/MS	Shellfish	[38]
MIL-101@Fe_3_O_4_	Magnetic MOFs	Phthalate esters	Fe_3_O_4_ NPs: 70	0.08–0.15 μg/L	MSPE	GC–MS	Human serum	[31]
Fe_3_O_4_@Fe-BTC	Magnetic MOFs	Blood lipid regulators	9.7	4–99 g/L	d-SPE	UPLC-MS/MS	Water	[33]
Fe_3_O_4_ NP–modified 2D zinc MOF	Magnetic MOFs	Fluoroquinolones	Fe_3_O_4_ NPs: 10–15	0.009–0.016 ng/mL	MSPE	HPLC–MS/MS	Milk	[34]
MOF-derived NH_2_-Fe_3_O_4_@C	Magnetic MOFs	Tramadol and methadone	10	73–32 pg/mL	MSPE	GC–MS	Urine samples	[37]
Magnetic UiO-66-NH@DES)	Magnetic MOFs	PFAIs	20	2.81–34.3 pg/g	MSPE	GC–MS	Edible oils	[35]
PCN-250(Fe_2_Mn) MOF + CoFe_2_O_4_ MNPs	Magnetic MOFs	EDCs	CoFe_2_O_4_ MNPs: 69.5	1.5–53 ng/L	SBSDME	LC–MS/MS	Urine	[36]
Fe_3_O_4_ @TbBd	Magnetic COFs	Estrogens	40	0.2–7.7 ng/L	MSPE	HPLC–MS	Human urine	[41]
COF-(TpBD)/Fe_3_O_4_	Magnetic COFs	Phthalate esters	50	0.005 to 2.748 μg/L	MSPE	GC–MS/MS	Beverage samples	[42]
Fe_3_O_4_@COF(TpBD)@Au-MPS	Magnetic COFs	Fluoroquinolones	Fe3O4 NPs: 100	0.1–1.0 μg/kg	MSPE	HPLC–MS/MS	Food samples	[43]
NH_2_– Fe_3_O_4_@COF	Magnetic COFs	Benzoylurea insecticides	NH_2_–Fe_3_O_4_ NPs: 50	0.06–1.65 ng/L	MSPE	UHPLC-MS/MS	Tea beverages	[44]
MCOF-composite	Magnetic COFs	Diclofenac sodium	Fe_3_O_4_@SiO_2_ NPs: 15	0.01–3.5 μg/kg	MSPE	HPLC–MS	Milk	[45]
MNP@COF	Magnetic COFs	Aristolochic acid I	40	1.84–2.21 ng/L	MSPE	HPLC–MS/MS	Mouse serum and tissues	[46]
NiFe_2_O_4_/COF	Magnetic COFs	Quinolones	NiFe_2_O_4_ NPs: 50–100	0.09–0.26 μg/L	MSPE	UHPLC-MS	Animal innards	[47]
Fe3O4@silk fibroin@COFs	Magnetic COFs	Sulforaphane	Fe_3_O_4_ NPs: 10	0.5 ng/mL	MSPE	HPLC–MS/MS	Cruciferous vegetables	[48]
Fe_3_O_4_@COF-on-COF	Magnetic COFs	Sulforaphane	15.2	0.052–0.092 μg/L	M-DSPE	HPLC–MS/MS	Cruciferous Vegetables	[49]
Fe_3_O_4_@NH_2_ − MIL-125@TpPa-SO_3_H	MOF@COF hybrid	Quinolones	Fe_3_O_4_ NPs: 50	0.0016–0.0940 ng/g	MSPE	HPLC–MS	Meats	[50]
C_16_mimBr -MCNTs@SiO_2_	MCNT	Flavonoids	Fe_3_O_4_ NPs: 100	0.20–0.75 ng/mL	SPE	HPLC–MS	Human urine	[72]
GO-Fe_3_O_4_ nanocomposite	Magnetic graphene NPs	Psychoactive drugs	Fe_3_O_4_ NPs: 2–10	0.02–0.2 μg/L	MSPE	UHPLC-MS/MS	Human urine	[57]
Fe_3_O_4_@SiO2–G	Magnetic graphene NPs	Pesticides and pollutants	15–40	0.005–0.030 ng/g	MSPE	GC–MS	Tomato and rape	[58]
Fe_3_O_4_@GO	Magnetic graphene NPs	Sulfonamides	< 100	0.02–0.013 μg/L	MSPE	HPLC–MS/MS	Milk samples	[59]
MGO	Magnetic graphene NPs	2-glycerol monopalmitate	< 100	-	d-SPE	GC–MS	Olive oil	[60]
GO/Fe_3_O_4_	Magnetic graphene NPs	Nitroimidazoles	Fe_3_O_4_ NPs: 10–20	0.003–0.08 μg/kg	MSPE	UPLC-MS/MS	Honey	[61]
Fe_3_O_4_/rGO	Magnetic graphene NPs	PAHs	< 100	0.02–14.3 ng/L	MSPE	GC–MS	Water	[62]
Mag-MIP	MIP	Sterols	Fe_3_O_4_ NP: 30–50	80.00 μg/L	MSPE	GC–MS	Mushroom, human serum, watermelon	[51]
CF@m-CNTs-MIP	MIP	Catecholamines	Fe_3_O_4_ NPs:30–50	0.023–0.054 μg/L	d-μ-SPE	UFLC-MS/MS	Human plasma	[52]

*CE-* Capillary electrophoresis, *COF* covalent-organic frameworks, *EDCs* endocrine disrupting compounds, *d-SPE* dispersive solid-phase extraction, *GAs* gibberellic acids, *MOF* metal–organic frameworks, *MIP* molecularly imprinted polymers, *MNPs* magnetic nanoparticles, *MSPE* magnetic solid-phase extraction, *NPs* nanoparticles, *PAHs* polycyclic aromatic hydrocarbons, *PFAIs* perfluoroalkyl iodides, *SPE* solid-phase extraction, *VOCs* volatile organic metabolites

### Removal of interfering proteins

Crude extracts for metabolite analysis are typically complex mixtures that contain a wide range of metabolites along with macromolecular components such as DNA, RNA, proteins, and lipid bilayers that can compromise both broad metabolomic profiling, and the targeted isolation, detection, and accurate quantification of individual metabolites [7]. Among them, proteins are particularly problematic, as they distort the baseline spectra and interfere with mass spectrometry signals, hindering accurate metabolite profiling. Conventional methods to remove protein background typically rely on ultrafiltration or precipitation using organic solvents [16]. An alternative strategy involves nanoparticle-based pretreatment that leverages electrostatic and hydrophobic interactions between nanomaterials and protein surface residues. In particular, anionic silica NPs (SNPs) with an average diameter of 20 nm were used to selectively bind proteins in human serum, promoting their aggregation and co-precipitation. The best results were obtained when the SNP addition to serum samples was combined with either ultrafiltration or an organic-solvent based approach. Nuclear magnetic resonance (NMR) was used to evaluate the signal quality after treatment with SNPs. This strategy effectively removes serum proteins from the matrix, facilitating downstream metabolite analysis [17]. Such approaches enhance metabolite detection sensitivity by minimizing macromolecular interference without extensive sample manipulation. While these sample methods primarily target protein removal, recent strategies have shifted toward the selective enrichment of target metabolites, aiming to both reduce interference and improve detection the sensitivity and efficiency of mass spectrometry (MS) identification of trace compounds.

### Nanoparticle-based strategies for selective metabolite enrichment

Limited sample volumes and the inherently low abundance of many compounds in complex biological matrices pose major challenges for accurate metabolite detection and quantification. These factors significantly impact the sensitivity, reproducibility, and accuracy of downstream LC–MS and LC–MS/MS analyses. Therefore, sample preparation must not only remove interfering matrix components but also enable selective enrichment of target compounds. Nanoparticle-based extraction strategies have gained attention to improve analyte recovery, enhance detection sensitivity, and mitigate matrix effects [18].

Solid-phase extraction (SPE) remains a widely used technique that relies on the reversible adsorption of analytes onto a stationary phase from a solution, followed by elution with a suitable solvent [19]. The integration of nanoparticles into SPE workflows has attracted attention due to their high surface-area-to-volume ratios, versatile surface chemistry, and tunable affinity for diverse metabolites. Advanced nanomaterials, such as metallic and metal oxides NPs, magnetic nanoparticles (MNPs), metal–organic frameworks (MOFs), covalent organic frameworks (COFs), carbon nanoparticles (CNPs), and molecularly imprinted polymers (MIPs), have been developed as selective and efficient SPE sorbents [15]. In parallel, SPE formats have evolved to enhance analytical performance, throughput, and automation. Techniques such as magnetic SPE (MSPE), solid-phase microextraction (SPME), stir-bar sorptive extraction, dispersive SPE (d-SPE), and flow-based automated SPE have been widely adopted. These configurations benefit directly from the physicochemical versatility of nanostructured sorbents, which improve metabolite selectivity, enrichment capacity, and compatibility with complex sample matrices. Downstream analytical workflows typically rely on separation and MS-based detection using techniques such as GC–MS, GC–MS/MS, GCxGC-MS, UHPLC-MS, and UHPLC-MS/MS.

Beyond general preconcentration, functionalized NPs offer selectivity toward specific metabolite classes. Surface modifications with ligands, functional groups, or biomimetic coatings enable selective interactions with specific chemical functionalities. Table 1 further highlights the remarkable versatility and specificity of NP-based sorbents across a wide range of metabolite classes and sample types. NP-assisted SPE has been successfully applied to recover low-abundance metabolites from complex matrices, such as biological fluids (human plasma, urine, serum), environmental samples, and food matrices (milk, tea, honey, beverages, meat). This approach been effectively applied to isolate diverse metabolites, which include aldehydes, amines, amino acids, amino metabolites, antioxidants, carbamates, catecholamines, estrogens, flavonoids, ginsenosides, ketones, lipids, monoamine neurotransmitters, mycotoxins, nucleosides, oxidized phospholipids, perfluorinated compounds, phenols, phthalate esters, sterols, and volatile organic compounds. Applications also extend to illicit drugs, pharmaceuticals, sedatives, pesticides, herbicides, insecticides, and other contaminants in environmental, biological, food, and food-packaging samples (Table 1). For Table 1 and all subsequent tables in this review, only studies employing nanoparticles strictly within the nanoscale range (all three dimensions < 100 nm) were included, whether as individual functionalized NPs or as core components of composite materials. Studies were excluded if, despite being referred to as NPs, the reported particle size exceeded 100 nm or if size data were unavailable. Notably, most studies focus on the enrichment of a few specific compounds or individual metabolites, typically ranging from single targets to over 60 analytes (e.g., [20–22]). This trend likely reflects the enhanced sensitivity and selectivity of nanoparticle-assisted SPE, which aligns more closely with targeted approaches than with the comprehensive profiling typical of untargeted metabolomics.

Notably, magnetic NPs (MNPs) continue to dominate due to their ease manipulation, reusability, and compatibility with MSPE formats [21–26] (Table 1). Their magnetic responsiveness enables rapid and efficient separation using external magnets, which eliminates the need for column packing, reduces solvent use, simplifies sample handling, and minimizes analyte loss. MNPs are also reusable, making them cost-effective and environmentally friendly [27]. Magnetic core–shell silica NPs with C_18_-modified surfaces (Fe_3_O_4_@SiO_2_-C_18_) has been proposed as a fast and efficient sorbents for the extraction and enrichment of low-concentration pyrethroid pesticides from aqueous samples, achieving limits of detection (LOD) as low as 0.001 µg/mL by employing GC–MS, and enrichment factors up to 1015-fold [24]. A similar Fe_3_O_4_@SiO_2_-C_18_ composition was also applied for the selective isolation of urinary volatile organic compounds (VOCs) associated with cancer. In that study, GC–MS analysis yielded low LODs (9.7 ng/mL), good accuracy (75–99%), and high precision (intra-day < 3%, inter-day < 11%). Compared to conventional sorbents, this approach offered shorter adsorption and elution times, supporting its potential as rapid and sensitive tool for early cancer detection [25].

The majority of MNPs studies listed in Table 1 targeted fewer than 10 metabolites, focusing on analyte classes such as pesticides, catecholamines, VOCs, and selected ginsenosides [23–26]. However, two studies highlight the critical role of boronate-affinity functionalization and polyethyleneimine (PEI) grafting in expanding metabolite selectivity, particularly for cis-diol-containing metabolites and glycosylated natural products [21, 22]. In the first, Li et al. [21] developed Fe_3_O_4_@SiO_2_PEI-FPBA nanoparticles for the selective enrichment of low-abundance ribosylated metabolites. The high density of boronate groups and improved solubility led to a six- to sevenfold increase in adsorption capacity over conventional boronate adsorbents, along with rapid enrichment (< 2 min). Sixty ribose conjugates were captured from human urine and detected by UHPLC-MS, which included nine novel modified nucleosides, demonstrating strong potential for biomarker discover [21]. In a similar approach, Zhao et al. [22] synthesized Fe_3_O_4_@PEI@TBA MNPs for capturing trace ginsenosides from rat plasma under neutral conditions, without protein precipitation. The PEI-assisted multivalent boronate interaction enabled a high absorption capacity and enhanced specificity. Coupled with HPLC–MS, this method identified 63 ginsenosides, significantly more than the 37 detected using conventional methanol extraction [22].

Metallic NPs, which include metal NPs and metal oxide NPs, possess unique optical properties, large surface energies, plasmonic excitation, and quantum confinement, which have been exploited for selective enrichment of metabolites [28]. For example, hybrid TiO_2_-ZrO_2_ (1–2 nm size) coated on porous silica (TiO_2_-ZrO_2_/SiO_2_) was developed as promising alternative to traditional boronate sorbents, for the selective enrichment of cis-diol nucleosides from urine via Lewis acid–base interactions [20]. The hybrid material demonstrated high capacity and selectivity even in the presence of a 500-fold interference. It enabled the identification, by HPLC–MS/MS, of nine nucleosides and 42 ribosylated metabolites, including two previously undetected boronate-based materials. These findings position it as a promising alternative to traditional boronate sorbents for cis-diol metabolite capture. and enabled the discovery of novel urinary biomarkers [20]. Similarly, gold NPs functionalized with hydrazide groups enriched aldehyde-containing oxidized phospholipids from human plasma, yielding up to 20-fold increase in detection sensitivity by LC–ESI–MS/MS [29].

MOFs have gained importance in sample preparation due to their tunable pore structures and surface chemistries, which are optimal for conducting adsorption, separation, and analyte enrichment. They have been applied as sorbents for removing impurities or concentrating target compounds and are often coupled with chromatography and mass spectrometry techniques to improve analytical performance. Furthermore, functionalization and hybridization of MOFs with nanoparticles or polymers can improve their stability and broaden their applicability [15, 30]. Particularly magnetic MOFs, appear frequently in Table 1, underscoring their growing importance. Their ability to adsorb a wide range of metabolites, such as phthalates, gibberellic acids (GAs), fluoroquinolones, endocrine disrupting compounds (EDCs), perfluoroalkyl iodides (PFAIs), and polycyclic aromatic hydrocarbons (PAHs) [31–36], and specific metabolites including domoic acid, tramadol, and methadone [37, 38], is attributed to their tunable porosity, large surface area, and ability to engage in multiple non-covalent interactions, such as hydrogen bonding, π–π stacking, and electrostatic forces, which facilitate the selective adsorption of analytes [39].

For instance, a magnetic MIL-101(Cr) nanocomposite was synthesized by decorating Fe₃O₄ nanoparticles onto MIL-101(Cr), prepared hydrothermally without using corrosive hydrofluoric acid, for extracting phthalate esters (PEs). The nanocomposite demonstrated excellent performance in DMSPE from plasma and environmental water samples, followed by GC–MS analysis. Key advantages of the method include high extraction efficiency, rapid extraction dynamics, reduced solvent and sorbent use, and strong interactions (hydrophobic, π–π, and coordination) facilitating effective analyte adsorption. The approach also offered low detection limits, good reproducibility, and a wide linear range [31]. Bagheri et al. [34] synthesized a 2D MOF (Fe_3_O_4_ NP–modified 2D zinc MOF) for extraction of fluoroquinolones from milk. A key implementation was the use of a 3d-printed microchip with a magnetic 2D MOF-filled microcolumn, and acetic acid was used as green eluent to avoid toxic solvents typically used in C18 cartridges. Coupled with LC–MS/MS, the method showed high sensitivity, low LOD (< 0.009 ng/mL), and strong selectivity, and demonstrated efficacy for food safety analysis [34]. A novel stir bar sorptive dispersive microextraction (SBSDME) method combined with LC–MS/MS was developed for detection of 12 EDCs in urine. For the first time, a stable magnetic MOF, PCN-250 (Fe_2_Mn), was used for analytical applications and, importantly, the method used a simple physical mixture of the MOF and cobalt ferrite (CoFe_2_O_4_) MNPs, eliminating the need for composite preparation. The method was efficient for EDC extraction, demonstrating the practical potential of this simplified MOF-MNP system [36].

For another hand, magnetic COFs also feature prominently. COFs are crystalline, porous materials with customizable pore sizes and surface chemistries, suitable for gas adsorption, separation, catalysis, and sample preparation. Their tunable stability has increased their use as adsorbents in SPE, SPME, DSPE, and MSPE methods, especially when combined with magnetic NPs, MOFs, and polymers [15, 40]. As shown in Table 1, have been applied in combination with MNPs, for enriching estrogens, phthalate esters, quinolones, fluoroquinolones, benzoylurea insecticides, diclofenac sodium, aristolochic acid I, and sulforaphane, in matrices such a as food and beverages, and human and mouse biofluids [41–48]. Fe_3_O_4_@silk fibroin@COFs and Fe_3_O_4_@COF-on-COF have been successfully applied for the extraction and analysis of the bioactive compound sulforaphane from cruciferous vegetables. The biomimetic magnetic Fe_3_O_4_@silk fibroin@COF was developed using Fe₃O₄@silk fibroin as the core and COFs grown via interfacial directional self-assembly. This multilayer material features a high surface area, large pore volume, and superparamagnetism, making it ideal for sulforaphane extraction. Adsorption occurs mainly through multilayer chemisorption, involving electrostatic forces, π-stacking, and hydrogen bonding. Applied to cruciferous vegetables, the method achieved up to 92% extraction efficiency in 30 min, with recoveries exceeding 73% [48]. Notably, for another hand, a magnetic COF-on-COF (Fe_3_O_4_@COF-on-COF) was developed using Fe_3_O_4_ as a magnetic core. The layered COF-on-COF structures, with their interlaced pores and varied shell thickness enhance selectivity and adsorption performance. This applied to extract sulforaphane from cruciferous vegetables through MDSPE and HPLC–MS/MS, enabled rapid magnetic separation (1 min), high extraction efficiency (up to 97%), and quick adsorption/desorption (5.5 min). Compared to monolayer COFs and conventional methods, this approach is faster, more sensitive, solvent-efficient, and reusable for at least seven cycles, which highlights its strong potential for high-throughput sulforaphane analysis in food samples [49]. The MOF@COF hybrid (Fe_3_O_4_@NH_2_ − MIL-125@TpPa-SO_3_H) was used as MSPE adsorbent for simultaneous extraction of 17 trace quinolones residues in meats [50]. Combined with HPLC-Q-Orbitrap HRMS, the method showed excellent linearity (*R*^2^ ≥ 0.9978), low detection limits (0.0016–0.0940 ng/g), and high precision (RSD < 5.8%). It was applied to spiked pork, chicken, and beef, and achieved recoveries of 83.9–106.2%. The high selectivity was attributed to π–π interactions, hydrogen bonding, and electrostatic attraction between QNs and the functional groups of the composite.

Carbon-based nanomaterials such as magnetic multi-walled carbon nanotubes (MWCNTs) and molecularly imprinted polymers (MIPs) have shown promising applications in selective enrichment of trace sterols and catecholamines due to their high binding capacities and tailored surface architecture [51–53]. These nanomaterials enable efficient isolation of specific metabolite subclasses, supporting downstream identification and quantification, even in complex biological samples. This targeted approach improves the analytical sensitivity and reduces background noise and matrix effects, which is particularly beneficial for studying specific biomarkers or metabolite subclasses in challenging samples types [54–56].

As shown in Table 1, magnetic graphene–based nanomaterials, those integrated with nanoparticles in the context of this review, integrated with nanoparticles, have demonstrated effective performance in selectively enriching compounds such as psychoactive drugs, pesticides, pollutants, sulfonamides, nitroimidazole residues, and PAHs in complex samples like urine, milk, honey, and water. When coupled with advanced analytical techniques such as HPLC–MS/MS and GC–MS, these materials offer high sensitivity and low detection limits [57–62]. Several of the reviewed studies further highlight the versatility of these systems. For instance, a GO-Fe_3_O_4_ nanocomposite synthesized by co-precipitation enabled extraction by MSPE of eight psychoactive drugs from urine with good linearity (up to 1000 µ/L), high recoveries (80–105%), and low LODs (0.02–0.2 µ/L) [57]. A silica-coated magnetic graphene composite (Fe_3_O_4_@SiO_2_-G) was synthesized to extract and enrich 14 pesticides from tomato and rape samples. The silica layer improved surface functionality and stability, which resulted in LODs as low as 0.005 ng/g and recoveries of up to 110.3% [58]. Similarly, F_e_3O_4_@GO was optimized for extraction of 15 sulfonamides from milk, achieving LODs of 0.02–0.13 µ/L and recoveries of 73–97% [59]. In quality control of olive oil, magnetic GO (MGO) was used in d-SPE to determine 2-glycerol monopalmitate (2-GMP), and demonstrated superior clean up efficiency compared to traditional silica gel sorbents [60]. A GO/F_e_3O_4_ nanocomposite synthesized via a simplified one-step co-precipitation method was employed for detecting nitroimidazole drugs and their metabolites in honey, with LODs of 0.003–0.08 µg/kg and recoveries of 66–90%, and demonstrated rapid preparation and minimal solvent use [61]. In environmental monitoring, the F_e_3O_4_/rGO nanocomposite synthesized by a solvothermal method was applied to selective extraction of polycyclic aromatic hydrocarbons (PAHs) from water samples. Its high adsorption efficiency allowed for the treatment of 100-mL water samples using just 10 mg of sorbent, yielding recoveries of 75.6–112.4% and LODs as low as 0.02 ng/L [62].

Moreover, molecularly imprinted polymers (MIPs), including Mag-MIP and COF@MWCNTs-MIP, incorporating Fe₃O₄ NPs (30–50 nm), have demonstrated high selectivity and achieved accurate quantification of sterols and catecholamines in complex matrices such as human plasma, serum, and mushroom extracts. Magnetic molecularly imprinted polymers (MIPs) incorporating Fe₃O₄ nanoparticles have shown excellent selectivity in complex biological matrices. In one study, β-sitosterol mag-MIP beads synthesized via microwave-assisted polymerization enabled the GC–MS detection of three sterols (ergosterol, stigmasterol, β-sitosterol) in mushrooms, serum, and watermelon, with up to 20-fold enrichment and recoveries of 71.6–88.2% [51]. In another, a carboxyl-functionalized magnetic carbon nanotube MIP (CF@m-CNTs-MIP) was developed for the extraction of catecholamines from plasma, showing high recoveries (87.5–110%) and LOQs as low as 10 ng/L, with successful application in clinical samples [52].

Nanoparticle properties are generally defined by their size, shape, structure, composition, surface characteristics, and functionalization [63]. Nanoparticle size plays a critical role in their interaction with biomolecules, influencing adsorption efficiency, surface reactivity, and biomolecular corona formation. Upon exposure to biological matrices, nanomaterials rapidly form a corona of proteins and small metabolites, which can alter their biological identity, affect uptake, distribution, and toxicity. While protein coronas have been widely studied, the metabolite corona, formed by low molecular weight compounds, has recently gained attention due to its significant impact on nanoparticle behavior. The composition and recovery of this corona depend on nanoparticle properties, such as size and surface chemistry, as well as on sample preparation conditions. Together, these factors affect the selectivity and sensitivity of NP-assisted metabolite analysis [64, 65].

Equally important is the recovery of metabolites bound to nanoparticles, which is critical for downstream analysis, such as MS-based analysis. Recovery efficiency depends on the nature of the nanoparticle–metabolite interaction and surface chemistry. High recovery rates have been reported using common elution strategies such as pH shifts, high-salt buffers, or organic solvents. For nanoparticles functionalized with cleavable affinity ligands (e.g., boronic acids, azobenzene), chemical cleavage, or competitive elution using excess ligand enables targeted metabolite release [23, 64, 66, 67]. However, the synthesis of functionalized nanoparticle for NP-assisted metabolite faces challenges due to the need for sensitivity, selectivity, reproducibility, and compatibility with complex biological matrices [15]. In this context, chemical synthesis remains the most widely used approach, offering efficiency, scalability, and low cost [68]. Compared to solid-phase synthesis methods, the fabrication of nanoparticles and nanostructures is generally more cost-effective [69]. The complexity of synthesizing functionalized nanoparticles increases with structural sophistication. While metallic NPs are relatively easy to synthesize and functionalize, achieving uniformity, stability, and balancing properties such as particle size and magnetic response time, remains a challenge [15]. More complex nanostructures, such as hybrid or porous frameworks, offer enhanced performance for metabolite capture but require advanced synthesis and functionalization protocols [39]. Ongoing efforts aim to simplify these processes and improve reproducibility to support broader integration of nanomaterials in metabolite analysis workflows.

Nanoparticles have become essential tools for sample pretreatment and metabolite enrichment, addressing challenges like macromolecular interference and low analyte concentrations. In general, once these nanomaterials are integrated into microextraction methods, they present advantages and simplify analytical workflows, reduce sample loss, and improve analytical performance compared to conventional SPE sorbents [70, 71]. Their unique physicochemical properties include (i) a high surface area-to-volume ratio, which enhances analyte interaction and extraction efficiency; (ii) tunable surface chemistry for selective functionalization; and (iii) better dispersion in complex matrices, particularly in dispersive formats, minimizing handling steps and potential losses [13, 71]. These advantages make nanomaterial-based sorbents powerful alternatives for simplifying and improving sample preparation workflows across diverse analytical contexts.

Ongoing advances in nanoparticle design are expected to further expand their utility in complex biological and environmental analyses. However, increased efforts are needed to translate these developments into real-world applications.

### Metabolite chemical modification/nanoparticles for targeted metabolite derivatization

In recent years, the integration of nanostructures and microparticles as chemoselective platforms, through the incorporation of derivatization functionalities onto their surfaces, has enabled selective interaction with metabolites based on their functional groups such as amines, carboxylic acids, thiols, hydroxyls, aldehydes, and ketones [67, 73–76]. The captured metabolites can then be released through cleavable linkers responsive to enzymatic activity, photochemical stimuli, or chemical agents like acids or reductants [77, 78].

The versatility of these probes lies in their capacity to simultaneously enrich and derivatize target analytes. This dual function enhances ionization efficiency, chromatographic resolution, and significantly reduces background interference from complex biological matrices. Additionally, derivatization tags often generate diagnostic MS fragment ions, aiding in the identification of unknown or low-abundance metabolites [13]. To date, only a limited number of studies have applied nanoparticles in a strict sense (structures with all dimensions in the 1–100 nm range) for direct derivatization purposes. Among the few true nanoparticle-based derivatization systems, two notable examples stand out, highlighting the potential but underexplored scope of this strategy. A notable example is a chemo-selective nanoprobe based on mesoporous silica nanoparticles (mSiO₂@azobenzene–COOH) developed for profiling amino metabolites in complex biological samples. The probe-integrated azobenzoic acid reacts with amino groups, which enables the selective capture of amino metabolites (Table 2). It also serves as a cleavable linker, easily broken down by sodium dithionite, and as a derivatization agent. The attached benzene ring and tertiary amine enhance chromatographic retention and MS sensitivity, significantly improving detection of amino metabolites in biological matrices [67].

**Table 2:** Typical applications of nanoparticles in the pretreatment of biological samples for targeted metabolite derivatization

Nanoparticles	Size	Metabolites	LOD	Analytical instrument	Samples	Reference
mSiO_2_@azobenzene–COOH	mSiO2 NPs: 70 nm	Amino metabolites	20–500 pg/mL	UHPLC-MS	Human serum	[67]
AuNPs/[Ph3PAu]3O + BF4ˉ	< 30 nm	Neurotransmitters	-	MALDI-TOF–MS	Mice brain tissue extracts	[76]

Another example involves a novel in situ derivatization strategy using tris(triphenylphosphine) gold oxonium tetrafluoroborate AuNPs/([Ph_3_PAu]_3_O^+^BF_4_^−^) for rapid MALDI-TOF–MS detection of neurotransmitters in mouse brain tissue extracts. This approach promotes the formation of gold nanoclusters (Au NCLs) through aerophilic interactions with neurotransmitters, which lead to enhanced UV absorption and ionization efficiency. It enables simultaneous derivatization and detection of multiple neurotransmitters (including dopamine, noradrenaline, serotonin, GABA, histamine, and tyramine) with a tenfold faster response than conventional pyrylium-based matrices [76]. These studies demonstrate the promising but still nascent application of NPs for metabolite derivatization, particularly at the true nanoscale.

Recent advancements in nanoparticle-assisted sample preparation have underscored the important role of targeted derivatization in metabolomics. Functionalized, cleavable probes on nanostructures enable simultaneous enrichment and chemical modification of diverse metabolite classes, improving ionization, sensitivity, and structural identification. Whether through azobenzoic acid–tagged silica or gold-mediated in-situ derivatization, these strategies demonstrate how precise chemical modification is essential to unlocking low-abundance or chemically challenging metabolites from complex matrices.

## Nanoparticle-enhanced chromatographic separation of metabolites

### Nanoparticles in stationary phases: application in capillary electrophoresis and liquid chromatography

Chromatographic separation is a crucial step before MS analysis to ensure metabolites are well separated, avoiding peak overlap and matrix effects. Recently, various nanoparticles such as silica (SiO₂) NPs, metal NPs, MWCNTs, MOFs, COFs, carbon dots, and graphene oxides (GO) have been introduced as stationary or pseudostationary phases to enhance metabolite separation (Table 3). These nanoparticles increase interaction sites, introduce new separation mechanisms, and improve the selectivity for diverse metabolites [79–82].

**Table 3:** Applications of nanoparticle-based stationary phases for chromatographic separation

Nanoparticles	Size (nm)	Phase	Metabolites	Separation-detection technique	Ref
AuNPs	15	Stationary	Hydrophobic alkylbenzenes	CEC-UV	[84]
AuNPs	15	Stationary	Hydrophobic, polar and basic compounds	CEC-DAD	[85]
AgNPs	40–80	Stationary	Sterols, fatty acid methyl esters, tocopherols, and polyaromatic hydrocarbons	CEC-DAD	[88]
AgNPs	5–10	Stationary	Aromatic hydrocarbons	HPLC–UV	[86]
Au-Fe_3_O_4_	100	Stationary	Dihydroxy benzene isomers	CE-UV	[87]
AuBNNT	AuNP: 10	Stationary	Benzene and naphthalene derivatives	HPLC–UV	[93]
AuNPs/GO	20	Stationary	Amino acids, nucleosides, and nucleobases	HPLC–UV HPLC-ELSD	[92]
Sil-Glc-CDs	Glc-CDs: 4.5	Stationary	Amino acids, saccharides, ginsenosides, nucleosides, and nucleobases	HPLC–DAD	[91]
NCDs	3.5	Stationary	Nucleosides, nucleobases, saccharides	HPLC–UV	[96]
GQDs	16–27	Stationary	Alkaloids, nucleosides, nucleobases	HPLC–UV	[90]
PPDCDs	5	Stationary	Amino acids, sugars, ginsenosides	HPLC–UV	[82]
ImCDs	3	Stationary	Bases, nucleosides, amino acids, saccharides, ginsenosides	HPLC–UV	[97]
Glc-NCDs	2.45	Stationary	Amino acids, ginsenosides, saccharides, nucleoside and base	HPLC–UV HPLC-ELSD	[89]

*BNNT* Boron nitride nanotubes, *PPDCDs* phenylenediamine-based carbon dots, *ImCDs* imidazolium ionic liquids-derived carbon dots. Nitrogen-doped, glucose-based carbon dots. *GQDs* graphene quantum dots

In capillary electrophoresis (CE) and capillary electrochromatography (CEC), nanomaterials are often used in three ways: dispersed in the background electrolyte as pseudostationary phases, adsorbed on the capillary wall as semi-permanent phases, or covalently bonded as permanent phases. This versatility helps to regulate electroosmotic flow (EOF) and strengthens metabolite interactions, improving separation efficiency, selectivity, and reproducibility [80, 83].

A variety of nanoparticles have already demonstrated enhanced chromatographic behavior when primary incorporated as stationary phases, especially in liquid chromatography (Table 3). Taking advantage of their strong π–π interactions and surface plasmon resonance, AuNPs and AgNPs, ranging in size of 5–80 nm, have been commonly applied for separating hydrophobic alkylbenzenes, aromatic hydrocarbons, polycyclic compounds, and dihydroxy benzene isomers [84–87]. In addition, larger AgNPs have been applied for the separation of more complex non-polar analytes, such as tocopherols and polyaromatic hydrocarbons [88].

According with reports listed in Table 3, carbon-based nanoparticles, particularly carbon dots (CDs) such as nitrogen-doped CDs [89], graphene quantum dots (GQDs) [90], an glucose-based CDs [91], typically 2.5–5 nm in size, have demonstrated efficiency for separating polar compounds such as amino acids, nucleosides, nucleobases, and saccharides. Hybrid materials, such as AuNPs on graphene oxide (AuNPs/GO) [92] and boron nitride nanotube–supported AuNPs (AUBNNT) [93], broaden the range of detectable metabolites and add tunable surface properties. Most of these nanoparticle-based phases are compatible with HPLC coupled with UV or ELSD detectors, with high resolution separation demonstrated across different metabolite classes. Carbon dots and their derivatives dominate as functional stationary phases for metabolite separation in HPLC–UV workflows, whereas AuNPs have been primarily employed in CEC-UV or CEC-DAD systems, which show their utility in the analysis of non-polar and aromatic compounds.

Despite their promise, the integration of NP-assisted separation with MS detection remains limited. Few studies have demonstrated seamless and compatible coupling with MS workflows. However, some promising examples exist using nanomaterials, although not particles in the strict sense. For instance, Tong et al. [94] developed a graphene-embedded poly(BMA–EDMA) monolithic capillary column for polymer monolith microextraction (PMME) of glucocorticoids in cosmetics, coupled to HPLC–MS. Graphene significantly improved column loading capacity, achieving low detection limits and high recoveries [94]. In other study, the same group synthesized a GO/graphene nanosheet-coated poly(GMA-EDMA) monolith, for the selective extraction of sarcosine, a biomarker of prostate cancer. By using HPLC–MS/MS, this strategy achieved a 32-fold enrichment enhancement and recoveries of ~ 93% [95]. These remain among the few successful examples of nanostructured materials integrated with MS-based workflows.

## Nanoparticle-assisted detection and ionization for metabolite analysis

### Enhanced ionization in mass spectrometry: MALDI, SALDI, and LDI-MS platforms

Matrix-assisted laser desorption/ionization mass spectrometry (MALDI-MS) is widely used for rapid detection and spatial localization of biomolecules, relying on organic matrices such as 2,5-dihydroxybenzoic acid (DHB), α-cyano-4-hydroxycinnamic acid (CHCA), 3,5-dimethoxy-4-hydroxycinnamic acid and 9-aminoacridine (9-AA). However, these matrices present limitations that include low ionization efficiency, uneven co-crystallization with analytes, ion suppression, and significant background noise, especially for metabolites below *m/z* 500 due to matrix interference [98]. To overcome these issues, a variety of nanomaterials (metals, metal oxides, MOFs, COFs, and carbon-based particles) have been developed as alternative matrices (Table 4), offering minimal background interference, high salt tolerance, efficient desorption/ionization, and superior thermal and chemical stability, thus improving sensitivity and reproducibility [99, 100]. Matrix-free approaches such as laser desorption/ionization mass spectrometry (LDI-MS) and surface-assisted LDI-MS (SALDI-MS) also benefited from nanomaterial integration, improving reproducibility, reducing background noise, and improving the detection of low molecular weight (LMW) metabolites [101]. These platforms support both targeted metabolite analyses and comprehensive metabolomics, and along with LDI, MALDI, and SALDI, have been successfully applicated in mass spectrometry imaging (MSI) for spatially resolved molecular analysis (Table 4) [102].

**Table 4:** Applications of nanoparticle-based matrix for LDI-MS, SALDI-MS, and MALDI-MS detection

Nanoparticles	Size (nm)	Metabolites	LOD	Technique	NP Class	Detection mode	Reference
COF-V@Au	AuNPs: 20	Metabolomics	-	LDI-MS	COFs	Positive	[124]
CTO@Au	AuNPs: < 100	Metabolomics	-	LDI-MS	Metal oxides	Positive	[125]
Au@SiO_2_@ZrO_2_	85	Metabolomics	-	LDI-MS	Metal oxides	Positive	[123]
FeOOH@ZIF-8	ZIF-8 NPs: 50	Metabolomics	-	LDI-MS	MOFs	Positive	[133]
MOF/Pt	PtNPs: 8–10	Metabolomics	-	LDI-MS	MOFs	Positive	[134]
Bimetallic MOF-NPs	Zn-Co NPs: < 100	Metabolomics	-	LDI-MS	MOFs	Positive	[135]
ZrMOF/Au	87.7	Metabolomics	-	LDI-MS	MOFs	Positive	[126]
^109^ Ag and AuNPs	< 100	Carboxylic acids	Ag NPs: 13–13,056 ng/mL; AuNPs: 1625–19,562 ng/mL	LDI-MS	Noble metals	Positive	[113]
^109^ Ag	< 100	LMW compounds	-	LDI-MS	Noble metals	Positive	[103]
Au	30	LMW compounds	82 nM	LDI-MS	Noble metals	Positive/negative	[106]
Au nanoshells	AuNPs: 14	Amino acids, carbohydrates	3–30 pmol	LDI-MS	Noble metals	Positive	[107]
SiO_2_@Ag	AgNPs: 4	Metabolomics	-	LDI-MS	Noble metals	Positive	[112]
^109^ Ag	< 100	Amino acids	0.4–300 ng/mL	LDI-MS, LDI-MSI	Noble metals	Positive	[121]
Au@MSN@Ag	AuNPs: 45	LMW compounds	5–100 fmol	LDI-MS	Noble metals	Positive/negative	[111]
Ag nanostructures	AgNPs: 50	LMW compounds	-	LDI-MS	Noble metals	Positive/negative	[114]
PdAu@Au	50	Metabolomics	10–50 µmol/L	LDI-MS	Noble metals	Positive	[108]
TiO_2_/MXene	TiO_2_ NPs: 42	Metabolomics	-	LDI-MSI	Metal oxides	Positive	[131]
PdPtAu	Au/Pd/Pt NPs: 50/30/10	Metabolomics	-	LDI-MSI	Noble metals	Positive	[127]
CDs	2–5 nm	Oligosaccharides, amino acids, fatty acids	0.2–20 fmol	MALDI-MS	Carbon-based	Positive/negative	[136]
N,S-CDs	2–3	LMW compounds	-	MALDI-MS	Carbon-based	Negative	[137]
Bi2O3@GO	Bi_2_O_3_ NPs: 50	LMW compounds	**-**	MALDI-MS	Carbon-based	Negative	[138]
Fe_3_O_4_/ZrO_2_	100	Oxidized phospholipids	200 pM	MALDI-MS	Metal oxides	Positive/negative	[129]
MoS_2_/Ag	AgNPs: 9	Amino acids, fatty acids	0.5–5 pmol	MALDI-MS	Noble metals	Negative	[128]
GDs	6	Oligosaccharides	0.3–0.75 fmol	MALDI-MSI	Carbon-based	Positive/negative	[139]
Fe_3_O_4_/WO_3_/Ag	Fe_3_O_4_/WO_3_/Ag NPs: 11/65/20	Metabolomics	-	MALDI-MSI	Metal oxides	Positive/negative	[130]
Ag	6 nm	Lipids	-	MALDI-MSI	Noble metals	Positive/negative	[118]
PVP-capped Ag	AgNPs: 3.84	Lipids	0.5–5 μM	MALDI-MSI	Noble metals	Positive	[119]
AgNPs@PDA	95	Glycerophospholipids and sphingolipids	-	MALDI-MSI	Noble metals	Positive/negative	[120]
Au/GDY	AuNPs: 52	Sulfacetamide and LMW compounds	-	SALDI-MS	Carbon-based	Positive	[122]
Fe_3_O_4_	39	Amino acids, carbohydrates, nucleosides	1030 μM	SALDI-MS	Metal oxides	Positive	[132]
Au	13	Urinary biomarkers	1.5–1.8 μM	SALDI-MS	Noble metals	Positive	[104]
Au	7	Amino acids	20–30 pmol/μL	SALDI-MS	Noble metals	Positive	[105]
Au@GMSN	70	LMW compounds	0.05–10 pmol	SALDI-MS	Noble metals	Positive	[109]
(AgNP/rGO)_9_	AgNP: 30	LMW compounds	-	SALDI-MS	Noble metals	Positive/negative	[110]
MTF-AuNP	AuNP: 13.7	Hydroxyproline	1–100 μM	SALDI-MS	Noble metals	Positive	[115]
^109^AgNPs	< 100	Mycotoxins	-	SALDI-MS	Noble metals	Positive	[116]
AuNPs	60	Crystal violet molecules	-	SALDI-MS	Noble metals	Positive	[117]

*SLDI* Soft laser desorption/ionization mass spectrometry, *HMOHs* hierarchical metal oxide heterojunctions, *TMOHs* trimetallic oxide heterojunctions, *pSi* porous silicon, *NAPA* nanopost arrays, *TCSI-MS* tip-contact sampling/ionization, *HCNFs* homogeneous carbon nanosphere film-spot, *Co-NC* Co-incorporated mesoporous carbon material, *CNF* carbon nanofibers, *GDY* graphdiyne

As shown in Table 4, noble metals such as silver (Ag) and gold (Au), and alloys are widely used due to their strong UV–Vis absorption under and thermal conductivity, which facilitate efficient energy transfer and analyte desorption. As well as being the most used in LDI-MS and SALDI-MS-based approaches [103–117], maybe the techniques where they provide the most advantage, due to matrix-free desorption and high sensitivity. Nobel metal nanoparticles present versatility across metabolite classes such as metabolomics-wide approaches, amino acids, carboxylic acids, carbohydrates, lipids, glycerophospholipids, and other analytes like sulfacetamide, urinary metabolites and dyes. Silver nanoparticles (AgNPs) have been used in MALDI-MSI analyses for in situ brain lipid imaging after mild traumatic injury [118]. In this context, it has been demonstrated that polymer-capped AgNPs (polydopamine or polyvinylpyrrolidone) enhance metabolite interaction and reduce the interference from silver cluster ions, broadening detectable lipids classes including low-abundance glycerophospholipids and sphingolipids [119, 120]. Furthermore, monoisotopic AgNPs simplify isotopic patterns in LDI-MS platform and increase sensitivity and quantification limits [103, 121]. Gold nanoparticles (AuNPs) have shown better performance in detecting polar and ionic metabolites, such as nucleosides, saccharides, amino acids, and LMW polymers via SALDI-MS [104, 105, 109, 115, 122] and LDI-MS [106, 107, 123–126]. Au nanoshells generate hot carriers more efficiently than nanorods or spheres, enhancing the detection sensitivity of amino acids and carbohydrates in complex samples with interfering human serum proteins [107].

Noble metals such as gold and silver can also be incorporated into alloy or composite nanostructures to improve LDI-MS performance. Alloy nanoparticles, like mesoporous PdPtAu alloys [127] and PdAu@Au concave cubes [108], offer tunable plasmonic properties and efficient hot carrier generation. While composite structures such as Au@GMSN [109], AgNPs-rGo films [110], MoS_2_/Ag hybrids [128], and Au@MSN@Ag nanohybrids [111] combine plasmonic metals with functional support to enhance analyte interaction, laser absorption, and signal reproducibility.

Transition metal oxides, with strong UV absorption and low heat capacity, also facilitates metabolite ionization [123, 125, 129–132]. Modified Fe₃O₄ and zirconia NPs enabled enrichment and sub-nanomolar detection of oxidized phospholipids, by leveraging electrophilic interactions and derivatization strategies [129]. Similarly, multi-matrix combinations integrating Fe₃O₄, tungsten oxide, AgNPs, DHB, 9-AA, and 1,5-diaminonaphthalene have achieved the visualization of nearly 600 metabolites in germinated corn seeds, including glycolysis and TCA cycle intermediates [130].

Recent trends indicate a shift from targeted to broader untargeted metabolomics using nanoparticle-assisted LDI-MS, MALDI-MS, and SALDI-MS, reflecting growing demand in clinical and biological applications [108, 112, 123, 127, 130]. Nanoparticle matrices markedly improve ionization efficiency, reduce background interference, and broaden detectable metabolite classes, by addressing the limitations of traditional organic matrices. Looking forward, the development of more stable, tunable nanomaterials with controlled energy transfer promises improved sensitivity, selectivity, and spatial resolution. Nanoparticle-based matrices thus provide powerful platforms for sensitive, reproducible, and comprehensive metabolite profiling, opening new avenues for biological and clinical research.

## Conclusions and future perspectives

The application of MS-based metabolite analysis has rapidly advanced as a fundamental research field for understanding biological processes, enabling both comprehensive metabolomic profiling and focused quantification of specific metabolites across spatial and temporal dimensions.

Progress in analytical instrumentation, sample preparation, and data analysis, particularly the integration of bioinformatics, has significantly expanded metabolite coverage, especially for low-abundance compounds in small-volume samples. Among these innovations, nanoparticle-based strategies have emerged as powerful tools for enhancing the selectivity of metabolite detection in complex biological matrices.

Nanoparticles, both as discrete entities and as components of nanocomposites or hybrid materials, offer remarkable advantages. Their large surface area, tunable surface chemistries, and unique optical, magnetic, and thermal properties improve sample enrichment, derivatization, separation, and ionization. These enhancements have enabled the detection of diverse and analytically challenging metabolites, which includes those with poor ionization efficiency, high polarity, or low abundance, benefiting both untargeted metabolomic studies for biomarker discovery and targeted quantification of individual or specific metabolite classes. One of the common threads across the reviewed studies is the efficacy of structured nanoparticle composites in nanoparticle-assisted SPE workflows. Their high surface area and selective interactions with analytes, through hydrogen bonding or π-π stacking, contribute significantly to enhanced recovery and selectivity, making them ideal for metabolite enrichment in complex matrices.

In the ionization phase of MS workflows, NP-assisted methods such as MALDI, SALDI, and LDI have enhanced sensitivity, specificity, and reproducibility of MS-based detection. Noble metals, particularly gold and silver improve ionization efficiency through plasmonic effects, enabling better detection of a broader range of metabolites with reduced background noise. Advanced designs such as core–shell architecture and hybrid nanomaterials further promote efficient energy transfer and plasmon-induced hot carrier generation, amplifying analytical performance. MOFs and COFs, as matrices, combine structural tunability with high proton transfer capacities and serve as promising matrix alternatives for both profiling and MSI.

Despite these promising advances, challenges remain for NP-assisted metabolite analysis. If considered for routine use, it is worth noting that while nanoparticles offer many advantages, it remains uncertain whether they may lead to gradual residue accumulation, highlighting the need for proper optimization and careful maintenance. Suboptimal selectivity and recovery in nanoparticle–based sorbents, the complexity of synthesizing chemoselective probes, and the stability of nanoparticle-coated stationary phases can limit routine applicability. In MALDI and related MS techniques, in-source fragmentation (ISF) of metabolites and the formation of multiple ions adducts can compromise quantification and identification accuracy. Future improvements addressing these issues will depend on designing more precisely engineered nanoparticles, a deeper mechanistic understanding of NP-analyte interactions, and tighter integration with advanced bioinformatic pipelines. Surface functionalization strategies that allow targeted chemical derivatization on nanoparticles promise to selectively enhance detection of specific metabolite classes. Although challenging, these chemoselective modifications are a promising avenue to overcome limitations in analyte recovery and specificity.

As nanoparticle-assisted metabolomics continues to evolve, the development of multifunctional, highly selective nanomaterials tailored to specific metabolite classes is expected to further expand the analytical capabilities of MS-based platforms. Furthermore, integration with advanced imaging, single-cell analysis, and AI-driven data interpretation is expected to yield insights across biological scales and increase the translational impact of metabolomic research.

The development of multifunctional, stable, and precisely engineered nanoparticles tailored to specific metabolite classes will be essential. These improved designs are expected to enhance selectivity, robustness, and reproducibility by leveraging surface chemistry modifications and enhanced understanding of nanoparticle–analyte interactions. Efforts toward seamless integration of nanoparticle enrichment with chromatographic separation, ionization, and advanced data analysis pipelines, including automation and miniaturization, will be critical to achieving high sensitivity and throughput while reducing sample and reagent consumption. To ensure reproducibility and routine applicability, establishing standardized protocols for nanoparticle synthesis, characterization, and application is imperative. Benchmarking and interlaboratory studies will help validate these methodologies across diverse biological matrices.

Looking forward, the focus is shifting toward more robust, reusable, and eco-friendly coatings, supported by computational tools and machine learning to optimize nanoparticle design for specific tasks. To fully realize the potential of nanoparticle-assisted metabolite analysis, emphasis must also be placed on the scalability and cost-effectiveness of nanoparticle synthesis and functionalization processes. Developing stable, reusable nanomaterials will not only enhance economic viability and sustainability but also support their broader application. Coupling nanoparticle-based enrichment with chromatographic separation prior to MS detection offers improved selectivity and reduces matrix effects, facilitating smoother integration into MS workflows. These advances are critical for enabling rapid, high-throughput analysis in different areas such as clinical diagnostics, environmental monitoring, and food safety. Ensuring that such methodologies are accessible, adaptable, and commercially viable will be essential for their routine implementation in diverse analytical settings.

In summary, nanoparticle-assisted strategies are transforming the landscape of MS-based metabolite analysis by increasing sensitivity, selectivity, and analytical workflow efficiency. Whether applied to untargeted profiling or the precise quantification of specific biochemical targets. Moving forward, interdisciplinary collaboration will be essential to overcome current challenges and fully unlock the potential of nanotechnology in analytical science.

## Data Availability

No datasets were generated or analysed during the current study.
